# Prognostic Analysis of Patients with Acute Myocardial Infarction Undergoing Implantation of Different Stents for the First Time

**DOI:** 10.3390/jcm10215093

**Published:** 2021-10-29

**Authors:** Cheng-Chung Cheng, Fang-Han Yu, Pi-Shao Ko, Hsiao-Ting Lin, Wei-Shiang Lin, Shu-Meng Cheng, Sui-Lung Su

**Affiliations:** 1 National Defense Medical Center, Department of Internal Medicine, Division of Cardiology, Tri-Service General Hospital, Taipei City 114, Taiwan; allexlll@gmail.com (C.-C.C.); fishhanhan0303@gmail.com (F.-H.Y.); 2 National Defense Medical Center, School of Public Health, Taipei 114, Taiwan; kimirrarike@gmail.com (P.-S.K.); tommy1129d@yahoo.com.tw (H.-T.L.)

**Keywords:** bare-metal stent, drug-eluting stent, ST-elevation myocardial infarction, non-ST-elevation myocardial infarction, major cardiovascular incident

## Abstract

For patients with acute myocardial infarction scheduled to undergo percutaneous coronary stent implantation, in most cases a drug-eluting stent is recommended as the first choice for treatment. However, there is a lack of research on the effectiveness of bare-metal stents and drug-eluting stents on patients with different types of myocardial infarction. Our objective was to explore the effects of bare-metal stents and drug-eluting stents on patients with different types of myocardial infarction in terms of major cardiovascular incidents. This retrospective cohort study included 934 patients with myocardial infarction undergoing coronary artery stent implantation for the first time at the cardiac catheter room of the Tri-Service General Hospital in the Neihu District between 2014 and 2018. Patients’ information, including demographic data, laboratory data, cardiac echocardiography results, and angiocardiography results, was collected by reviewing medical records. Cox proportional hazards regression was used to adjust the potential confounding factors, and the adjusted data were then used to compare the correlation between different types of stents and major cardiovascular incidents in patients with ST-elevation myocardial infarction and non-ST-elevation myocardial infarction. After the confounding factors were adjusted, in patients with ST-elevation myocardial infarction receiving a drug-eluting stent compared with those receiving a bare-metal stent, it was found that the mortality risk was lower in terms of all causes of death (Adj-HR = 0.26, 95% CI = 0.14–0.48, *p* < 0.001) and cardiogenic death (Adj-HR = 0.20, 95% CI = 0.08–0.55, *p* = 0.002), the risk of non-fatal myocardial infarction was lower (Adj-HR = 0.17, 95% CI = 0.04–0.73, *p* = 0.017), and there was no difference in the risk of revascularization at the lesion site (Adj-HR = 0.59, 95% CI = 0.24–1.43, *p* = 0.243). It terms of the findings in patients with non-ST-elevation myocardial infarction, those receiving a drug-eluting stent had a lower risk of revascularization at the lesion site (Adj-HR = 0.48, 95% CI = 0.24–0.97, *p* = 0.04); however, there was no difference in the mortality risk in terms of all causes of death (Adj-HR = 0.71, 95% CI = 0.37–1.35, *p* = 0.296) or cardiogenic death (Adj-HR = 0.59, 95% CI = 0.18–1.90, *p* = 0.379),or in the risk of non-fatal myocardial infarction (Adj-HR = 0.27, 95% CI = 0.06–1.25, *p* = 0.093). Compared with bare-metal stents, drug-eluting stents provide better protection against death to receivers with ST-elevation myocardial infarction; however, this protection is decreased in receivers with non-ST-elevation myocardial infarction. It is recommended that for patients with non-ST-elevation myocardial infarction who are indicated to receive a drug-eluting stent, the clinical effectiveness of the treatment must be considered.

## 1. Introduction

Patients with acute myocardial infarction (AMI) are currently mainly treated with percutaneous coronary intervention (PCI). Compared with bare-metal stents, drug-eluting stents can decrease the chance of reinfarction; however, they are more expensive, and their receivers are subject to a longer period of medication and are more likely to experience complications, including intracranial hemorrhage and gastrointestinal bleeding [1]. Bare-metal stents are less expensive, but they come with a higher risk of reinfarction, which limits their clinical utilization [2]. Because different complications following interventions have different mechanisms, studies aiming at a single prognosis have shown inconsistent results. Thus, further and more comprehensive discussion is needed to examine the advantages and disadvantages of these two kinds of stents. AMI can be categorized into ST-elevation myocardial infarction (STEMI) and non-ST-elevation myocardial infarction (NSTEMI). Compared with patients with NSTEMI, those with STEMI usually have fewer comorbidities [3,4,5,6,7,8]. The atheroma in patients with STEMI, red thrombus, is mainly formed by fibrous protein aggregation, which is suitably treated using medications for thrombolysis. On the contrary, the atheroma in patients with NSTEMI, white thrombus, is mainly formed by blood platelet aggregation [9,10,11,12,13]. These two forms of thrombus have different pathological mechanisms, which should be treated using different methods. To treat STEMI, in most cases only the present conditions should be considered; however, to treat NSTEMI, the influences from comorbidities should be considered as well. The benefits of using drug-eluting stents in patients with STEMI are apparent. However, patients receiving a drug-eluting stent are required to undergo a longer period of dual antiplatelet therapy, which can increase the possibility of an interaction between the medication and other comorbidities rather than STEMI [14]. Thus, this study assumed that, for patients with NSTEMI and complicating comorbidities, drug-eluting stents could adversely lead to more major cardiovascular incidents.

Previous studies have found that bare-metal stents and drug-eluting stents showed inconsistent outcomes regarding major cardiovascular incidents. Mahmoud et al. and Mauri et al. have reported that drug-eluting stents, compared with bare-metal stents, could lower the risk of major cardiovascular incidents [3,15]. In contrast, in the studies by Yin et al., Gao et al., and Piscoine et al., no difference was noted between these two kinds of stents regarding major cardiovascular incidents [16,17,18]. Further, according to the study by Kastrati et al., no difference was found between bare-metal stents and drug-eluting stents in terms of the mortality risk to patients with AMI [19]. On the other hand, Sabaté et al. suggested that drug-eluting stents could be more beneficial in lowering mortality risk than bare-metal stents [20]. This study found that the subjects in past research that showed significantly better protection in drug-eluting stents were younger and had fewer comorbidities. In contrast, those in the research showing an insignificant difference had more comorbidities. This finding confirmed our study hypothesis; yet, there is a lack of direct evidence to compare the difference between these two kinds of stents for patients with STEMI and those with NSTEMI. This cohort study conducted in one medical center aimed to analyze the risk of major cardiovascular incidents in patients with AMI who underwent coronary angioplasty with stent insertion for the first time over bare-metal stents and drug-eluting stents within two years of their operation. Further, this study also compared these two kinds of stents regarding their risks of major cardiovascular incidents in different types of MI.

## 2. Materials and Methods

### 2.1. Subjects

This study was a retrospective cohort study. The analyzed subjects were patients who underwent coronary angiography at the cardiac catheter room of the Tri-Service General Hospital in the Neihu District from January 2014 to December 2018. This study was approved by the Tri-Service General Hospital IRB (No. C202005133). The data used in this study were obtained from the cardiac catheter room of the hospital, and the data were processed to anonymize them from their owners for privacy purposes.

**Figure 1:**
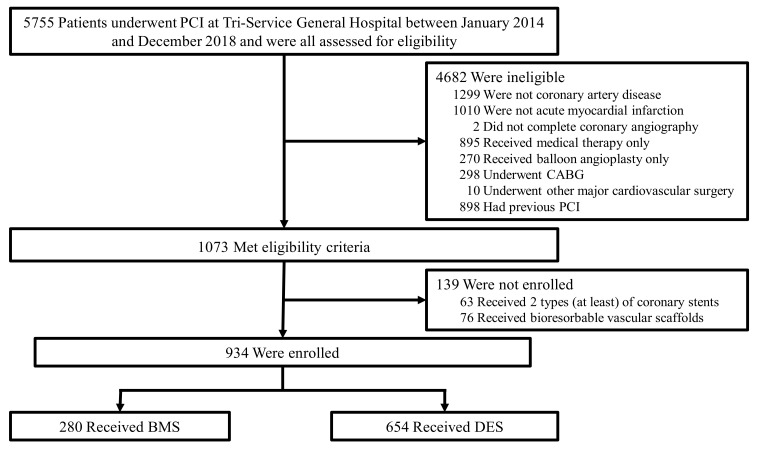
The flowchart for patient selection. BMS, bare-metal stent; CABG, coronary bypass graft surgery; DES, drug-eluting stent; PCI, percutaneous coronary intervention.

The flowchart of subject recruitment is presented in Figure 1. Subjects who met the following exclusion criteria were excluded: a diagnosis of non-coronary artery disease, no completed coronary angiogram, receiving only medication or undergoing only percutaneous transluminal coronary angioplasty (PTCA), undergoing major cardiovascular surgery within 2 weeks (valve repair or replacement, aortic aneurysm repair, or heart replacement), having a previous diagnosis of coronary artery disease, non-AMI, having ≥2 kinds of stents, or having a bioresorbable vascular scaffold. Finally, 934 subjects were included and divided into the bare-metal stent group and the drug-eluting stent group.

### 2.2. Study Endpoints and Definitions

The endpoints of this study were as follows: death from all causes, cardiogenic death, non-fatal MI, ischemic stroke, and target vessel revascularization. The diagnosis of AMI followed the clinical guidelines from the ACC/AHA, ESC and Taiwan Society of Cardiology, which has the following definitions: symptoms of myocardial ischemia, an electrocardiogram (ECG) indicating myocardial ischemia, a blood test with a troponin level higher than the limit of 99%. All subjects were categorized into STEMI and NSTEMI based on the abnormality of their ECG [21,22].

All PCIs were operated via patients’ femoral or radial artery. Before operation, patients were administered a loading dose of dual antiplatelet therapy, including 300 mg of aspirin, 600 mg of clopidogrel, or 180 mg of ticagrelor. During the operation, 70–100 IU/kg of unfractionated heparin was injected to maintain the activated clotting time over 250 s. Whether GPIIb-IIIa was administered was determined by the operating physicians. During hospital admission, the subjects were continuously administered dual antiplatelet therapy and other cardiovascular medications, such as beta-blockers, angiotensin-converting enzyme inhibitors or angiotensin receptor blockers, and hypolipidemics. Subjects continued to take the same medications after they were discharged.

The choice of a bare-metal stent or a drug-eluting stent was determined by the physicians who performed the operations. The types of drug-eluting stents available during the study were sirolimus, zotarolimus, everolimus, and Biolimus. There were no restrictions on the types or brands of the stents; that is, physicians could consider any of the four based on their consideration.

### 2.3. Data Collection and Management

Data was obtained by reviewing medical records to collect subjects’ demographic data, laboratory data, cardiac echocardiography results, and angiocardiography results. The basic demographic data collected included age, sex, smoking history, body height, and body weight. Body mass index (BMI) was calculated using body height and body weight, and obesity was defined as BMI ≥ 27. The comorbidities defined in this study included hypertension, diabetes, hyperlipidemia, stroke, heart failure, end-stage renal disease, and peripheral arterial obstruction disease. The laboratory data used included hemoglobin levels and glomerular filtration rate obtained on the same day or within 3 days postoperatively, as well as fasting blood glucose, total cholesterol, high-density lipoprotein, low-density lipoprotein, and triglyceride levels. The results of the cardiac echocardiography used in this study were those obtained within 3 months after the subject underwent coronary artery stent insertion for the first time, and only the data of left ventricular ejection fraction was used. The presence of multiple sites of vascular lesions, defined as ≥2 coronary arteries involved with different types of angiopathy, was determined based on the results of angiocardiography.

### 2.4. Statistical Analysis

SPSS 22.0 and R 3.5.1 statistical analysis software were used in this study to analyze the data collected from the day of stent insertion for the first time to investigate the occurrence of any of the clinical endpoints within 48 months postoperatively. The categorical variables in the subjects’ demographic characteristics were described using frequency distribution and percentages, and the continuous variables were described as mean ± standard deviation. To compare the demographic characteristics in different subject groups, Pearson’s chi-squared test was used for categorical variables, and independent samples *t*-test was used for continuous variables. In terms of the cumulative incidence rate of different stents, the Kaplan–Meier estimator and the log-rank test were used to describe the data collected in the follow-up period. For outcome analysis, the Cox proportional hazards model was used to calculate the hazard ratios of each of the endpoint incidents of the two stents, with the confidence interval (CI) set at 95%. Subsequently, stratification was introduced based on the types of MI. Then, the Cox proportional hazards model was employed again to calculate the hazard ratios of the confounding factors before the data was corrected to those after correction.

## 3. Results

### 3.1. Distribution of Basic Data

The basic demographic characteristics of the included subject groups are presented in Table 1. Compared with those in the drug-eluting stent group, those in the bare-metal stent group had a higher average age and a higher percentage of patients with a smoking history, as well as a higher percentage of patients with a history of ischemic stroke or transient ischemic attack, signs of poorer left ventricle function, and lower average hemoglobin levels.

### 3.2. Analysis of Clinical Prognosis

**Figure 2:**
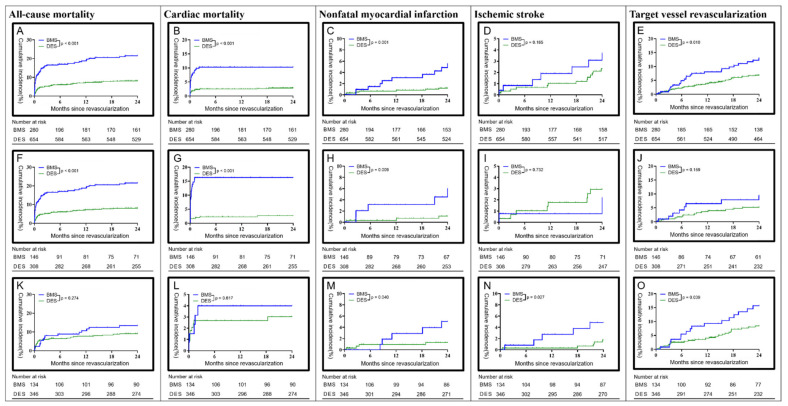
Cumulative 2 year incidence for patients with acute myocardial infarction. Kaplan–Meier estimates of major adverse cardiac events in: (**A**–**E**) all acute myocardial infarction patients; (**F**–**J**) ST-elevation myocardial infarction patients; (**K**–**O**) non-ST-elevation myocardial infarction patients. BMS, bare-metal stent; DES, drug-eluting stent.

The Kaplan–Meier estimator was used to calculate the cumulative incidence rate of major cardiovascular incidents within 2 years postoperatively. Compared with bare-metal stents, drug-eluting stents had a significantly lower cumulative incidence rate in terms of all causes of death, cardiogenetic death, non-fatal MI, and target vessel revascularization; however, no difference in the cumulative incidence rate of ischemic stroke was observed between the two groups (see Figure 2A–E). In subjects with STEMI, compared with bare-metal stents, those with drug-eluting stents had a significantly lower cumulative incidence rate in terms of all causes of death, cardiogenetic death, and non-fatal MI; no differences in the cumulative incidence rates of ischemic stroke and target vessel revascularization were observed (see Figure 2F–J). In subjects with NSTEMI, compared with bare-metal stents, those with drug-eluting stents had a cumulative incidence rate showing no difference in terms of all causes of death and cardiogenetic death; however, significantly lower incidence rates of non-fatal MI, ischemic stroke, and target vessel revascularization were observed (see Figure 2K–O).

### 3.3. Risk of a Major Cardiovascular Incident in Terms of Different Types of Coronary Artery Stents

The correlation between the types of stents and the occurrence of death and a major cardiovascular incident is shown in Table 2, which also presents further correlation analysis based on the types of MI. 

Regarding death, compared with bare-metal stents, those with drug-eluting stents had a lower mortality risk in terms of all causes of death (Adj-HR = 0.46, 95% CI = 0.24–0.85, *p* = 0.013). Among those with STEMI, those with drug-eluting stents had a lower mortality risk in terms of all causes of death (Adj-HR = 0.41, 95% CI = 0.18–0.93, *p* = 0.032); however, among those with NSTEMI, between the two kinds of stents, no significant difference was found in the mortality risk in terms of all causes of death (Adj-HR = 1.01, 95% CI = 0.39–2.63, *p* = 0.982). 

Those with drug-eluting stents had a lower risk of non-fatal MIs than those with bare-metal stents (Adj-HR = 0.10, 95% CI = 0.02–0.56, *p* = 0.009). However, among patients with STEMI and patients with NSTEMI, no significant difference was noted between the two kinds of stents.

Among patients with STEMI, no significant difference was found between the two kinds of stents in terms of target vessel revascularization (Adj-HR = 0.94, 95% CI = 0.23–3.83, *p* = 0.931); however, among patients with NSTEMI, those with drug-eluting stents had a lower risk of target vessel revascularization (Adj-HR = 0.36, 95% CI = 0.13–0.94, *p* = 0.037).

## 4. Discussion

This study found that, compared with bare-metal stents, drug-eluting stents could lower the risk of death in patients with MI, indicating a better protective effect. This finding was significant in the patients with STEMI (Adj-HR = 0.41, 95% CI = 0.18–0.93, *p* = 0.032) but not in those with NSTEMI (Adj-HR = 1.01, 95% CI = 0.39–2.63, *p* = 0.982).

Generally, to treat patients with AMI, drug-eluting stents are more effective than bare-metal stents. According to the randomized studies of EXAMINATION, COMFORTABLE AMI, and DEBATER, it has been found that, compared with bare-metal stents, drug-eluting stents are more effective in lowering the risk of major cardiovascular incidents in patients with AMI, which supports the finding of this study [20,23,24,25].

This study also found that drug-eluting stents could protect patients from ischemic stroke. This may be a benefit of drug-eluting stents because patients receiving a drug-eluting stent are indicated a longer period of thrombolytic drug use. Usually, patients with NSTEMI have more comorbidities and a higher risk of stroke, so the only benefit that a drug-eluting stent can bring is to lower the risk of ischemic stroke. In contrast, patients with STEMI have a lower risk of stroke and more severe damage to their heart, so the protective effect of drug-eluting stents against ischemic stroke is not significant. By analyzing the data of patients with NSTEMI regarding ischemic stroke, no difference was found between those with drug-eluting stents and those with bare-metal stents after correcting for comorbidities (Crude-HR = 0.29, 95% CI = 0.09–0.94, *p* = 0.039; Adj-HR = 0.16, 95% CI = 0.01–1.89, *p* = 0.147), which supports this hypothesis. In general, drug-eluting stents failed to bring significant benefit to patients with NSTEMI in terms of heart-related incidents.

This study found that drug-eluting stents could exert a protective effect on patients with STEMI, which is consistent with how these patients are currently treated with antithrombotic drugs [21,26,27]. The reason may be that the patients receiving a drug-eluting stent have taken antiplatelet drugs for a longer period, which decreases the occurrence of major cardiovascular incidents. In the clinical guideline, it has been mentioned that thrombolytic therapy is not recommended for those with NSTEMI [28]. Thus, patients receiving a drug-eluting stent, which includes a longer period of antiplatelet therapy, may suffer from more adverse effects, leading to no difference in the occurrence of major cardiovascular incidents compared with those receiving a bare-metal stent.

This study used data collected from patients who received their treatment before 2018. At that time, the second generation of drug-eluting stents required its receivers to undergo a long period of antiplatelet therapy. After 2019, when the third generation of drug-eluting stents became commonly available, patients are required to undergo a relatively shorter period of antiplatelet therapy [29,30,31,32]. Thus, further research to explore the effects of drug-eluting stents on patients with NSTEMI is warranted.

### Limitations

The limitations of this study may have exerted an influence on the results. First, all subjects were from one medical center, so a more variable sample is needed to verify the results of this study. In addition, the influence of different drug-eluting stents, differences in patients’ angiograms (e.g., the length of the lesion, the number of stents, the length of the stent, the inner diameter of the stent, and the operational details of the stent insertion), and relative medications on patients were not discussed in this study. Under the National Health Insurance System, the cost of a bare-metal stent is fully covered, but that of a drug-eluting stent is partially paid by patients, which can be a potential influencing factor behind what kind of stent is chosen. Additionally, patients’ personal insurance and socioeconomic status can play a role in deciding the stent.

## 5. Conclusions

Previous studies have found that drug-eluting stents can result in a better general prognosis in patients compared with bare-metal stents, but their effects on mortality risk remain equivalent. According to the studies on patients with AMI in Taiwan, drug-eluting stents can lower the mortality risk in these patients. This study further found that this protective effect was more significant in patients with STEMI, but not obvious in those with NSTEMI. However, this result still requires more randomized clinical trials to provide an evidence-based demonstration that bare-metal stents are effective in NSTEMI. When considering a drug-eluting stent in patients with NSTEMI, the economic benefits should also be considered. At present, drug-eluting stents have improved, and the third generation has been made available. In the future, further studies focusing on the analysis of different drug-eluting stents are warranted to provide more reference information in the clinical setting.

## Figures and Tables

**Table 1:** Basic demographic characteristics.

	Bare-Metal Stent (*n* = 280)	Drug-Eluting Stent (*n* = 654)	*p*-Value
Age, mean ± SD (years)	65.48 ± 15.07	62.83 ± 13.55	0.008
Male, *n* (%)	212 (75.7%)	532 (81.3%)	0.050
Obesity ^#^, *n* (%)	78 (28.7%)	203 (31.4%)	0.418
Smoking, *n* (%)	141 (50.4%)	272 (41.6%)	0.013
History of chronic disease, *n* (%)			
Hypertension	163 (58.2%)	388 (59.3%)	0.751
Diabetes	83 (29.6%)	207 (31.7%)	0.543
Hyperlipidemia	70 (25.0%)	172 (26.3%)	0.678
Ischemic stroke/TIA	39 (13.9%)	46 (7.0%)	0.001
Heart failure	22 (7.9%)	32 (4.9%)	0.075
End-stage renal disease	16 (5.7%)	43 (6.6%)	0.620
Peripheral artery obstructive disease	6 (2.1%)	6 (0.9%)	0.200
Laboratory data, *n* (%)			
Hemoglobin, mean ± SD (g/dL)	13.03 ± 2.87	13.92 ± 2.46	<0.001
GFR < 60 mL/min/1.73 m^2^	107 (38.2%)	210 (32.2%)	0.074
Impaired fasting glucose ^a^	239 (92.3%)	572 (91.2%)	0.610
Abnormal total cholesterol ^b^	141 (55.1%)	422 (67.0%)	0.001
Abnormal HDL cholesterol ^c^	102 (57.0%)	302 (58.0%)	0.818
LDL cholesterol, mean ± SD (mg/dL)	102.52 ± 36.70	109.42 ± 36.33	0.014
Abnormal triglycerides ^d^	33 (12.7%)	106 (16.6%)	0.146
LVEF < 40%, *n* (%)	45 (17.6%)	73 (11.7%)	0.021
Multiple sites of vascular lesion, *n* (%)	228 (81.4%)	501 (76.6%)	0.103
Type of MI, *n* (%)			0.157
STEMI	146 (52.1%)	308 (47.1%)	
NSTEMI	134 (47.9%)	346 (52.9%)	
Infarct-related artery, *n* (%)			
Left main artery	9 (3.2%)	52 (8.0%)	0.007
Left anterior descending	252 (90.0%)	590 (90.2%)	0.920
Ramus intermedius	9 (3.2%)	34 (5.2%)	0.185
Left circumflex artery	177 (63.2%)	421 (64.4%)	0.735
Right coronary artery	212 (75.7%)	482 (73.7%)	0.519
Extent of CAD, *n* (%)			0.081
1-vessel	52 (18.6%)	153 (23.4%)	
2-vessel	100 (35.7%)	190 (29.1%)	
3-vessel	128 (45.7%)	311 (47.6%)	
Stent			
Number, mean ± SD	1.73 ± 0.91	1.78 ± 1.01	0.407
Diameter, mean ± SD	3.05 ± 0.54	2.92 ± 0.43	0.002
Length, mean ± SD	24.20 ± 6.45	28.78 ± 9.66	<0.001
Discharge medication *n* (%)			
Anticoagulant	236 (84.3%)	629 (96.2%)	<0.001
β-blocker	195 (69.6%)	533 (81.5%)	<0.001
Calcium channel blocker	37 (13.2%)	111 (17.0%)	0.150
ACEI/ARB ^e^	148 (52.9%)	412 (63.0%)	0.004
Statin	186 (66.4%)	551 (84.3%)	<0.001
Antiarrhythmic agents	21 (7.5%)	58 (8.9%)	0.491

^#^ Obesity: BMI ≥ 27. ^a^ Fasting glucose > 100 mg/dL. ^b^ Total cholesterol > 150 mg/dL. ^c^ HDL cholesterol < 40 mg/dL. ^d^ Triglyceride > 200 mg/dL. ^e^ Angiotensin-converting enzyme inhibitor or angiotensin receptor blocker.

**Table 2:** Risk analysis of major cardiovascular incidents within 2 years.

	Cumulative Events (%)		Crude-HR (95% CI)	*p*-Value	Adj-HR (95% CI) ^#^	*p*-Value
	Drug-Eluting Stent	Bare-Metal Stent				
All patients with MI						
All causes of death	52 (8.0)	60 (21.4)	0.32 (0.22–0.47)	<0.001	0.46 (0.24–0.85)	0.013
Cardiogenic death	18 (2.8)	32 (11.4)	0.22 (0.12–0.39)	<0.001	0.46 (0.19–1.15)	0.096
Non-fatal MI	7 (1.1)	10 (3.6)	0.23 (0.09–0.60)	0.003	0.10 (0.02–0.56)	0.009
Ischemic stroke	13 (2.0)	8 (2.9)	0.54 (0.22–1.31)	0.172	0.63 (0.19–2.17)	0.467
Target vessel revascularization	38 (5.8)	24 (8.6)	0.52 (0.31–0.86)	0.011	0.51 (0.23–1.10)	0.086
STEMI						
All causes of death	21 (6.8)	44 (30.1)	0.18 (0.11–0.30)	<0.001	0.41 (0.18–0.93)	0.032
Cardiogenic death	8 (2.6)	27 (18.5)	0.12 (0.06–0.27)	<0.001	0.01 (0.00–3.92)	0.137
Non-fatal MI	3 (1.0)	5 (3.4)	0.18 (0.04–0.77)	0.021	0.22 (0.02–2.13)	0.191
Ischemic stroke	8 (2.6)	2 (1.4)	1.31 (0.28–6.20)	0.733	1.62 (0.15–17.64)	0.693
Target vessel revascularization	14 (4.5)	8 (5.5)	0.54 (0.23–1.29)	0.166	0.94 (0.23–3.83)	0.931
NSTEMI						
All causes of death	31 (9.0)	16 (11.9)	0.72 (0.39–1.31)	0.276	1.01 (0.39–2.63)	0.982
Cardiogenic death	10 (2.9)	5 (3.7)	0.76 (0.26–2.23)	0.618	256 (21.37–3072)	<0.001
Non-fatal MI	4 (1.2)	5 (3.7)	0.28 (0.07–1.03)	0.055	0.00 (0.00–6260)	0.735
Ischemic stroke	5 (1.4)	6 (4.5)	0.29 (0.09–0.94)	0.039	0.16 (0.01–1.89)	0.147
Target vessel revascularization	24 (6.9)	16 (11.9)	0.52 (0.28–0.98)	0.042	0.36 (0.13–0.94)	0.037

^#^ Age correction, sex, smoking history, ischemic stroke/TIA, hemoglobin, abnormal total cholesterol, LDL cholesterol, LVEF < 40%, left main artery infarct, stent diameter, stent length, anticoagulant, *β*-blocker, ACEI/ARB, and statin medication.
